# Current Mental Health Treatment Practices for Functional Neurological Disorders (FND): A Provider Survey

**DOI:** 10.1002/brb3.71465

**Published:** 2026-06-19

**Authors:** Amy E. Werry, Johanna Messerly

**Affiliations:** ^1^ Department of Neurology University of Texas Health Science Center At San Antonio San Antonio Texas USA; ^2^ The Glenn Biggs Institute for Alzheimer's and Neurodegenerative Diseases San Antonio Texas USA

## Abstract

**Objective:**

Functional neurological disorder (FND) conceptualization and treatment have rapidly evolved with mental health treatment representing a primary intervention target. Specific mental health treatment consensus recommendations have yet to be established as a standard of care. Clarifying current mental health treatment trends assists in programmatic planning while providing practical direction for clinical providers and researchers.

**Methods:**

A novel Qualtrics designed online survey was distributed to providers delivering FND mental health treatment services. Survey items addressed psychotherapeutic protocols, techniques, intervention strategies, and methods of determining treatment effectiveness and benefit. A total of 63 participants completed the survey from 12 countries, with the highest number being clinical psychologists/neuropsychologists in academic medical centers in the United States.

**Results:**

Variability was demonstrated in mental health treatment frameworks and modalities, though Cognitive Behavior Therapy (CBT) represented the most utilized, consistent with previous research. Specific therapeutic techniques included trigger chart/tracking of symptoms, symptom/episode response plan/health behavior monitoring and relaxation training. Daily functional status was the top post‐treatment outcome, exceeding patient report of symptom frequent/severity. Numerous barriers to implementation of FND mental health treatment were also described.

**Interpretation:**

This survey highlights the lack of consensus in mental health treatment for FND, including treatment implementation, outcome tracking, and defining successful outcomes. Future research is necessary to establish treatment guidelines via collaborative multi‐site trials as well as determine treatment effectiveness.

## Graphical abstract

This graphical abstract displays the primary results of the survey, which demonstrated variability in the implementation of mental health treatment frameworks, techniques, and modalities utilized across providers. Lack of consistency was observed in target treatment outcomes, contradicting most previously published trials in which the primary outcome variable was symptom frequency.

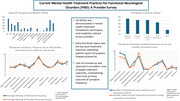

## 1 Introduction

The current diagnostic term of Functional Neurological Disorder (FND) is reflective of the most up to date understanding of etiology and symptom manifestation and is abbreviated from the American psychiatric Association Diagnostic and Statistical Manual of Mental Disorders (DSM; 2022) label of Functional Neurological Symptom Disorder. In the DSM, FND is diagnosed in the presence of altered motor or sensory functioning incompatible with a recognized neurological or other medical condition. The DSM lists several subtypes of FND, including weakness, abnormal movement, attacks or seizures, and mixed symptoms. Previous research has demonstrated the historical and cultural influences on the terminology, conceptualization, and understanding of symptoms that have resulted in recurrent updates and changes (Raynor and Baslet 2021), with the term “functional” being the newest iteration that has yet to be fully represented in research (Bratanov et al. 2023).

Overarchingly, there is a demonstrated need for multidisciplinary care that emphasizes breaking a cycle of emergent medical care during instances of acute or worsening FND symptoms. Patients with FND demonstrate high healthcare utilization (Beharry et al. 2021; Williams et al. 2022). Timely, targeted, and therapeutic delivery of the diagnosis is crucial in assisting patients bridge the gap from medical to “non‐medical” (Sireci et al. 2024). A recent study suggested a referral to a functional seizure specific treatment program alone diminished unproductive health care utilization (Bean et al. 2025). Further, rehabilitative services such as physical therapy/physiotherapy, occupational therapy, and speech and language therapy have consensus guidelines for FND treatment (Baker et al. 2021; C. Nicholson et al. 2020; Nielsen et al. 2015). There are no such consensus guidelines specifically for psychological/mental health treatment at present and research suggests diminished patient access to psychological treatment for FND (specifically functional/dissociative seizures [FDS]) and limited training for providers with diminished confidence in its implementation internationally (Areco Pico et al. 2025; Carter et al. 2018). Efforts are ongoing to address this need and guide treatment implementation in a multidisciplinary setting (Gutkin et al. 2021; Society 2024).

Mental health treatment is implicated as a necessary component of this multidisciplinary interventional framework (Sireci et al. 2024); Cope et al. 2025). Cognitive Behavioral Therapy (CBT) for FND, specifically FDS, has been effective in reducing number of episodes, and other symptoms of psychopathology, as well as improving reported quality of life (Goldstein et al. 2010; Goldstein et al. 2020; Lafrance et al. 2014; LaFrance et al. 2009). A systematic review indicated CBT was the most frequently used therapeutic modality in individual treatment for FND in general though psychodynamic therapy was also cited as promoting benefit (Gutkin et al. 2021). Other psychotherapeutic modalities and techniques such as mindfulness‐based therapy (MBT) for FDS (Baslet et al. 2020), motivational interviewing (Tolchin et al. 2020), short‐term psychodynamic therapy (Malda Castillo et al. 2022; Russell et al. 2022), acceptance and commitment therapy (ACT) (Graham et al. 2018), and validated trauma protocols such as Eye Movement Desensitization and Reprocessing (EMDR) and prolonged exposure (Myers et al. 2017; Cope et al. 2025) have also been cited as potential and promising intervention options.

Regarding the pediatric population, retraining and control therapy (ReACT) demonstrated significant reduction in FDS, using a CBT based framework (Fobian et al. 2020). A stress‐system model has also been proposed as helpful in conceptualizing FND symptoms and providing education in working with children and adolescents (Kozlowska 2017). A recent survey of pediatric providers in the United States demonstrated CBT was the primary psychotherapeutic approach (Watson et al. 2024).

Determination of successful treatment outcomes has also not been empirically solidified. Despite remission or decreasing symptoms, many patients continue to experience functional impairment at work, in home life responsibilities, as well as leisure activities (Goldstein et al. 2010; Goldstein et al. 2020; LaFrance et al. 2020; Tolchin et al. 2020). It has been argued that functional status may be a more important indicator for determining the success of psychological interventions for FND due to it having a strong relationship with perceived quality of life (Carlson and Nicholson Perry 2017; Goldstein et al. 2020; Tolchin et al. 2020; Varley et al. 2023). Other factors such as clinician perspective, illness duration, cognitive status, and concurrent psychotherapy have also recently been implicated in psychological treatment outcomes in outpatient settings (Bleier et al. 2025; LaFrance et al. 2023). While research has explored various treatments for FND and their impact on episode/symptom frequency and severity, there are still many unknowns about functional outcomes.

The current study sought to elucidate current mental health treatment practices for FND across a variety of settings. Determining present trends can assist in practice unification, collaboration to promote larger scale treatment validation, and determining standards for outcome measurement and evaluation of program effectiveness while ultimately moving toward establishing evidence‐based consensus recommendations.

## 2 Methods

### 2.1 Survey

An online survey was designed in Qualtrics (Provo, UT) by the authors and sent to professionals providing FND mental health treatment services. The survey was a novel instrument designed under the themes of individual providers’ choice and utilization of pscychotherapeutic protocols, techniques, intervention strategies, and methods of determining treatment effectiveness and benefit. Feedback on survey questions and breadth was elicited from professional mentors and experts in the field prior to dissemination. The survey included 30 questions, 12 of which were personal and professional demographic information. Questions were written in English, and all responses were anonymous. Questions were formatted using free text responses, multiple choice options, and ranked responses based on previous multiple choice selections. In an effort to be inclusive, questions allowed for selection of multiple responses whenever possible, which could have contributed to response overlap. This is not a previously used or validated questionnaire. Survey use and dissemination was approved by the institutional review board at the University of Texas Health Science Center at San Antonio (study number 874). The survey was accessible from the 31st of October 2024 to the 15th of April 2025.

### 2.2 Participant Inclusion

Inclusion criteria included providers who deliver mental health treatment services to patients with FND. There was no requirement for duration of professional practice, the type of mental health service, or licensure type/location. A link to the survey was sent to all members of several professional listservs, and professional contacts of known mental health treatment providers for patients with FND and was posted on the Functional Neurological Disorders Society (FNDS) research website. Listservs included special interest groups such as the FNDS Psychological Treatment SIG subspeciality organizations such as a neuropsychological listserv and target divisions of the American Psychological Association that are likely to have clinical experience with FND (rehab psychology and health psychology). Respondents were invited a single time within each listserv available to the authors. Information is not available on the number of respondents per recruitment strategy. The first question affirmed meeting inclusion criteria and consent to utilize data collected for research purposes.

### 2.3 Data Analysis

Data were exported from Qualtrics to SPSS Statistics v29 (www.IBM.com). All analyses represent simple means with standard deviation, totals, and sample percentages.

## 3 Results

Demographic information from participants is presented in Table 1. A total of 63 participants from 12 countries completed the survey. Within the US, there was a fairly even distribution across geographic regions. Representation from participants outside of the US was limited, with no representation from South America. There was a total of 19 participants who practice outside of the US. The majority of participants identified as female (n = 44, 69.84%). There were 10 different specialties represented, with the highest number of respondents identifying as clinical psychologists or neuropsychologists. 37 participants’ primary practice location was within an academic health center, 11 in a private outpatient clinic, 11 in a public hospital, and the other practice locations had few respondents represented. The majority of providers (n = 45) stated they provide services virtually and in‐person. The average years of practice experience was 12.41 (sd = 11.40), with a mean of 7.76 (sd = 8.01) years experience working with FND.

**TABLE 1:** Participant demographic and location data.

Demographic variable	N	Demographic variable	N
**Sex/gender**		**Practice setting**	
Male	16	Academic health center	37
Female	44	Armed forces medical center	1
DNS	2	Community health center	4
Gender	1	Community mental health center	1
		Community rehabilitation	1
**Country**		Private general hospital	6
Australia	3	Private outpatient clinic	11
Canada	3	Private psychiatric hospital	1
England	1	State/county/other public hospital	11
Egypt	4	VAMC	0
Israel	1	National UK service	1
Poland	1		
Scotland	1	**FND clinic type**	
South Africa	1	FND focused	12
Sudan	1	Component of broader practice	37
Netherlands	1	Multidisciplinary	23
UK	2		
United States	44	**Session setting/type**	
		In‐person	13
**US region**		Virtual	5
Northeast	12	Both virtual and in‐person	45
South	11	Individual only	33
West	8	Group only	2
Midwest	13	Both individual and group	21
			
**Specialty of provider participant**		**Age range of patients**	
Audiology	1	Adult	46
Counseling psychology	2	Pediatric	8
Clinical psychology	15	Lifespan	9
Clinical psychology and neuropsychology	2		
Neuropsychology	23		
Neurology	5		
Neurology and psychiatry	1		
Psychiatry	9		
OT	1		
Social work	3		
Stroke medicine	1		

Regarding practice characteristics (Tables 1 and 2), there was a mix of providers who offer services in FND focused clinics (n = 12), multidisciplinary clinics (n = 23), and as a component of a broader practice (n = 37). A large majority of participants offer services for adults only (n = 45), with few respondents working with pediatric (n = 8) and lifespan populations (n = 9). Participants reported collaborating with nine different provider specialties within their FND practice. Neurology was the most frequently endorsed, followed closely by neuropsychology and psychiatry. Two participants wrote in free text that they collaborate with school nurses or school representatives, and another included physiatry. Participants were most likely to endorse a monthly referral quantity of 0–5 referrals (n = 38).

**TABLE 2:** Practice location features.

	Mean	SD
**Provider experience**		
Total years practice (1‐55)	12.41	11.40
Years FND (1‐55)	7.76	8.01
What % practice is FND (2‐100)	34.98	30.0417
		
**Other involvement in sessions**	**N**	
Client/patient only	40	
Parent	23	
Spouse/significant other	24	
Other care partner	19	
		
**Most to least common duration rank for others (1–3)**		
Present full session (n = 45)	2.64	0.71
Present partial session (n = 45)	1.69	0.51
Encourage communication with support system (n = 45)	1.67	0.80
		
Typical wait time (weeks) (0‐99)	17.02	17.83
		
Number of sessions per patient (n = 54) (2‐60)	18.56	14.51
		
**Disciplines in collaboration**	**N**	
Counseling/psychology	33	
Social work	22	
Neuropsychology	41	
Neurology	47	
Psychiatry	38	
Speech therapy	27	
OT	26	
PT	28	
Schools	2	
		
**Referrals per month**	**N**	
0–5	38	
6–20	20	
21+	5	

For the mental health sessions (Tables 2 and 3), participants most frequently endorsed conducting sessions with the patient only; however, about 1/3 of participants endorsed including parents, care partners, and significant others in sessions. Those who involved other support people in sessions were most likely to encourage the person with FND to communicate with their support system or have the support person present for part of sessions as opposed to the full session. CBT was the most used therapeutic modality (n = 44), followed by psychoeducation (n = 39), mindfulness (n = 32), behavioral modification (n = 28), and ACT (n = 26). Participants were offered the option to write in other modalities utilized, and two mentioned “CFT” (this abbreviation was not clarified), two reported a combined or eclectic approach, and there were single mentions of emotional awareness and expression therapy, “sleep” interventions, and increasing physical activity. When asked about following protocols, 25 participants reported using *Taking Control of Your Seizures* (Reiter et al. 2015), 16 reported using Overcoming Functional Neurological Symptoms (Williams et al. 2017), and 11 endorsed using the protocol from the CODES trial (Goldstein et al. 2020). Free text responses included two mentions of a combination of multiple manualized programs and two reporting a customized or self‐created protocol. Other mentions included Children's Health and Illness Recovery Program by Carter and colleagues (Carter et al. 2020), CBT for Adults with Non‐Epileptic Seizures by Franny Moene, and Treating Somatic Symptoms in Children and Adolescents (Williams and Zahka 2017). Utilization of specific therapeutic techniques was widely varied (Tables 4 and 5). Cognitive restructuring was most endorsed, with 42 participants affirming use, and relaxation training was second most utilized with 40 users. At the other end of the spectrum, negative reinforcement was least reported and endorsed (3 participants). The majority of other techniques were utilized by 24–37 participants. Participants were asked to rank how frequently they use each therapeutic technique and how often they experience success using the technique, with low numbers representing most utilized and most frequently perceived as experiencing positive benefits. Aside from negative reinforcement, the range of average frequency of use was relatively small (3.70‐6.16) with large standard deviations, indicating a wide spread in frequency of use with no overtly consistent trends. The ranking of perceived success with the techniques was similar. Negative reinforcement stood out as the least perceived as successful, while the remaining range of average rankings was small with large standard deviations, again revealing no specific trends. Data included the number of participants who rated each therapeutic technique as their most frequently utilized and most successful. In looking at the data this way, trigger chart/tracking, cognitive restructuring, and symptom/episode response plans were most frequently ranked as most utilized; however, the range of ranking was still large. Similarly, symptom/episode response plans, trigger chart/tracking, and relaxation training received the most top rankings for most perceived success with a technique and also showed a large range of rankings.

**TABLE 3:** Practice implementation features.

Feature	N
**Therapeutic modality utilized**	
CBT	44
ACT	26
DBT	21
Mindfulness	32
EMDR	7
Psychodynamic	11
Emotion‐focused	8
Solution focused	13
Adlerian	0
Biofeedback	9
Gestalt	1
Hypnotherapy	3
Trauma‐focused CBT	12
Psychoeducation	39
Behavior modification	28
	
**Protocol used**	
Taking control	25
ReACT	7
CODES	11
Stress system model	6
Overcoming functional neurological Sxs	16
Patient workbook by FND Australia	4
	
**Assessments ordered/used**	
Patient report	52
Collateral report	42
Self‐report questionnaires (e.g., PHQ‐9, GAD‐7)	51
Objective psychological assessments (e.g., MMPI‐2, PAI)	30
Neuropsychological/cognitive assessment	40

**TABLE 4:** Therapeutic techniques used, frequency of use, and provider perceived success.

Technique	Number affirmative	Average ranking of utilization frequency scaled as most to least utilized (SD)	Average Ranking of Provider Perceived Success Scaled as Most to Least Successful (SD)
Trigger chart/tracking	33	4 (3.28)	3.71 (2.72)
Cognitive restructuring	42	3.70 (2.39)	4.44 (2.69)
Behavioral activation	37	5.37 (2.76)	4.96 (2.30)
Health behavior monitoring	24	4.50 (3.25)	5.33 (3.12)
Symptom/episode response plan	27	3.92 (3.06)	3.30 (2.40)
Positive reinforcement	23	5.74 (2.94)	5.33 (3.05)
Negative reinforcement	3	8.50 (0.71)	9.00 (0)
Exposure	25	5.40 (3.25)	5.11 (2.75)
Relaxation training	40	3.91 (1.78)	3.69 (2.19)
Grounding	37	4.09 (2.47)	4.06 (2.41)
Guided imagery	22	6.16 (2.57)	7.00 (2.74)
Thought record	27	5.28 (2.81)	5.74 (2.97)
Distraction	24	6.55 (2.82)	5.47 (3.26)
Other	5	4.75 (2.63)	3 (2.83)‐ 2 responses

**TABLE 5:** Therapeutic techniques ranked by frequency of use and provider perceived success.

Technique	Number of participants who ranked as most frequently utilized	Range of frequency ratings	Number rated as most frequently perceived as being successful in treatment	Range of success ratings
Trigger chart/tracking	9	1‐13	7	1‐10
Cognitive restructuring	8	1‐11	6	1‐11
Behavioral activation	1	1‐12	1	1‐9
Health behavior monitoring	4	1‐12	2	1‐12
Symptom/episode response plan	7	1‐10	9	1‐8
Positive reinforcement	0	2‐12	1	1‐12
Negative reinforcement	0	8‐9	0	9
Exposure	2	1‐12	2	1‐11
Relaxation training	3	1‐7	4	1‐11
Grounding	5	1‐9	4	1‐12
Guided imagery	0	2‐11	0	3‐11
Thought record	1	1‐13	2	1‐13
Distraction	1	1‐12	0	2‐12

In assessing severity of FND symptoms and treatment progress (Tables 3 and 6), simple patient report was most frequently utilized (n = 52), followed closely by self‐report questionnaires (n = 51). For determining treatment success, the spread of outcomes considered was tight. Symptom frequency was most utilized by 45 participants. Symptom severity and daily function status was reported by 41 participants each, and mood improvement was utilized by 34. Free‐text outcomes measured reported included quality of life, as well as the Quality of Life in Epilepsy questionnaire (Vickrey 1993), dissociation symptoms, physical outcomes, increased social interactions, PTSD symptoms, and how severely the person with FND is affected or bothered by symptoms. Following mental health treatment for FND, the participants ranked transition to general mental health treatment as the most common discharge status, followed by medical management in combination with general mental health treatment (Table 7).

**TABLE 6:** Therapeutic outcomes and frequency of use.

Outcomes	Number affirmative	Average ranking of utilization frequency scaled as most to least utilized (SD)
Symptom frequency	45	3.00 (1.13)
Symptom severity	41	2.51 (0.87)
Daily functional status	41	1.27 (0.65)
Mood improvement	34	3.03 (0.84)
Other	12	

**TABLE 7:** Provider rated discharge disposition frequency.

Discharge disposition (n = 46)	Average ranking of frequency encountered scaled as most to least	SD
General mental health therapy	1.78	0.87
No additional treatment	3.04	1.12
Medication management only	3.02	0.80
Med management and Gen MH therapy	2.15	1.12

Finally, participants were asked about barriers to intervention for FND and resources that would be beneficial for FND providers (Table 8). Reinforcement of symptoms by family or social support was the most highly endorsed perceived barrier to successful mental health treatment outcomes. Rejection of the diagnosis was the second most commonly endorsed barrier. Additional free‐text reports included avoidance, other medical symptoms, and other providers perpetuating incorrect diagnoses. The support that providers endorsed as most beneficial to their practice and outcomes was improved understanding of FND from referring providers. Increased access to other health disciplines, increased access to psychological treatment/tools, and peer consultation groups were endorsed as wanted resources by 32–33 participants.

**TABLE 8:** Provider perceived barriers to intervention and support needed for their practice.

Barrier/resource	N affirmative	Average ranking of frequency encountered scaled as most to least
**Barriers to intervention response**		
Insurance limited session number	10	5.98 (2.42)
Diagnosis rejection	39	2.29 (1.67)
Provider‐client alliance	11	5.50 (2.37)
Family/social support reinforcement of symptoms	41	3.36 (1.93)
Diminished health literacy	24	4.19 (2.38)
Transportation problems for in‐person	18	4.38 (1.71)
Technology problems for virtual	10	5.22 (2.22)
Inappropriate expectations by patient	27	3.33 (2.32)
Secondary gain	18	5.00 (2.16)
Cognitive/comprehension problems	21	3.82 (2.35)
Provider knowledge gaps	15	3.50 (2.18)
Provider experience gaps	20	2.89 (1.79)
Other	9	
		
**Support types would find beneficial**	**N**	
Increased access to other health disciplines	33	
Increased administrative support	23	
Improved understanding of FND from referring providers	43	
Increased access to psychological treatment/tools	32	
Peer consultation groups	33	

## 4 Discussion

The current study adds to growing research elucidating FND treatment trends, specifically in clarifying current mental health treatment practices for patients with FND across multiple countries and patient age bands. However, the majority of respondents were from the United States, with minimal representation from Asia and no South American representation. This highlights an ever‐growing need for international collaboration to optimize treatment practices. The majority of respondents indicated practicing in a variety of settings with the integration of FND treatment into a broader practice; few endorsed being in an FND specific practice. This is notable as previous research suggests the practicality of FND specific clinical services embedded within a larger neurology practice (Watson et al. 2024). Respondents endorsed varying years of experience in treating FND with variability in time devoted to FND clinical practice. In fact, FND mental health treatment did not account for even half of most of the respondents' clinical practice time, suggesting providers are managing multiple other clinical demands. This is notable given long wait times to establish care (mean = 17 weeks). This raises concern for patients who are told emergent care is not necessary when informed of the diagnosis but are restricted to an extended wait list. The majority of respondents provided care to adults only; thus, this represents a limitation to the pediatric population. Finally, the nature of responses suggests an intrinsic collaborative framework across multiple other disciplines such as medical (i.e., neurology, psychiatry) and rehabilitative (i.e., speech, physio/physical therapy). This is notable in that the majority of patients who complete an FND specific mental health treatment protocol are being referred for additional mental health treatment, suggesting FND providers need to have collaborations across numerous disciplines for optimal clinical services.

Pertaining to psychotherapy, many respondents endorsed utilizing a combination of individual and group settings. Group therapy has been less studied than individual protocols, though research indicates potential benefit (Barry et al. 2008; Boico et al. 2024; Chen et al. 2014). A group therapy option has numerous potential benefits, including diminishing wait time, improving access to care, and the additional therapeutic inclusion of peer support. However, this could limit implementation of individual specific psychotherapeutic techniques by necessitating generalization of a set therapeutic program to all members. Further, there raises potential for inadvertent reinforcement of the FND symptoms themselves and associated behaviors/responses (i.e., avoidance in refraining from active participation). Future research will be vital in further elucidating these concerns and exploring validation of either a standalone group therapy protocol or a hybrid with individual.

While CBT represented the most cited therapeutic modality, the spread of this was markedly varied, implying that mental health treatment providers are using a variety of frameworks. This is further illustrated in the highly endorsed use of cognitive restructuring, which is grounded in CBT principles (Beck 2021). Notably, the specific use of a thought record, which can be utilized for cognitive restructuring, was less endorsed and seen as less helpful. This, combined with trigger chart/tracking of symptoms, symptom/episode response plan/health behavior monitoring, and relaxation training was endorsed as the most frequently used and most successful technique. Several techniques that are specific to managing the FND symptoms themselves are not inherently specific to a psychotherapeutic framework. This suggests that practical management of the patients’ specific FND symptoms is seen as the most effective method of treatment. This is likely particularly helpful for individuals experiencing multiple FND subtypes. Marked variation and lack of consensus were also seen in the use of published FND specific protocols. This may highlight the need for increased flexibility in providing mental health treatment for patients with FND. Previous research has advocated for utilizing aspects of various evidence‐based treatment modalities to best meet patient needs (Myers et al. 2021). Results of the survey indicate this trans‐modality, as opposed to a modality or protocol specific, approach is being implemented. Future unifying research will be helpful in exploring pathways to solidify a treatment protocol with larger multi‐site validation studies that are replicated across differing cultural and linguistic contexts.

Regarding treatment outcomes/effectiveness, patient reports of symptom frequency and severity were endorsed with high frequency. General mood improvement was also noted, though less so. However, daily functional status was indicated as the top post‐treatment outcome. This is in the context of functional status not being explicitly defined for this survey. Oftentimes, improved functional status, while an important consideration for clinical practice, is not often the primary target outcome for a mental health treatment protocol in the research, but rather is the reduction of the target symptom (i.e., depressive symptoms as measured by a face‐valid/self‐report questionnaire). It also conflicts with the target outcomes of prior trials in which symptom (i.e., episode) frequency was the primary outcome of interest (Fobian et al. 2020; Goldstein et al. 2020; Lafrance et al. 2014; LaFrance et al. 2009). Thus, this demonstrates there is a lack of consensus on mental health treatment providers’ view of target outcomes for their practice. This is notable in light of ongoing efforts to identify and clarify treatment outcome measurement for FND, while affirming the nuance and complexity of establishing best practice (T. R. Nicholson et al. 2020).

Numerous barriers were cited to FND specific mental health treatment practice, including institutional/systemic (i.e., insurance limitations, transportation needs, diminished health literacy, secondary gain), intraprofessional (i.e., provider knowledge and experience gaps), and patient context specific (i.e., family support, diagnosis rejection, inappropriate expectations). These represent both domains of future research and opportunities to develop additional support to maintain an FND practice. Highlighting not only the need for collaboration and consultation opportunities across providers but also access to practical training on the implementation of FND treatment that is specific to the sociocultural context of the provider's area. It also suggests that recommendations specific to assessing, understanding, and navigating patient specific family systems and its influence on the FND symptoms themselves are a vital component of treatment. Broader efforts of advocating for FND mental health treatment specific services institutionally and within systems are also vital.

## 5 Limitations

There are notable gaps in regions represented among participants, with minimal to no representation in Asian, Sub Saharan Africa, and South American providers. This unfortunately follows other trends in research relying largely on representation from the global North, and any implications from the current survey should largely be discussed within those regions. Even within the global North, the highest representation was seen in the United States, which limits generalizability. Additional outreach and collaboration with mental health providers in missing regions could reveal different approaches and treatment outcomes that are more culturally congruent with their population.

While efforts were made to ensure opportunity for respondents to note information outside of the given responses, there are likely aspects of FND mental health treatment that were not fully represented in the survey. Further, mental health treatment practices specific to varying FND subtypes were not assessed in an effort to increase inclusivity. Future research will assist in delineating best mental health treatment practices for each FND subtype as the conceptualization of FND subtypes continues to evolve. This survey was also limited to professional listservs available to the authors and their connections, potentially resulting in the high representation of psychologists and neuropsychologists. Recruitment via listservs was a research design approach intending to ensure participants had appropriate inclusion credentials, but this strategy is not without faults. This survey's results and depth would be enhanced with broader representation from professional psychological organizations outside of the US in particular as well as additional disciplines.

## 6 Conclusion

The aim of the current study was to identify current trends in mental health practice for FND, with the goal being to inform not only the current state of practice but also to inform future treatment and outcomes research with an eye towards common definitions of successful management outcomes. Themes present in the survey results indicate providers are utilizing a variety of therapeutic frameworks and often a combination of frameworks, while practical symptom management appears to be perceived as the most beneficial strategy. However, there is a lack of consistency in mental health treatment providers’ view of target beneficial outcomes for their mental health treatment practice, which is contradictory to most trials defining outcomes by a single marker of symptom frequency.

## Author Contributions

**Amy E. Werry**: conceptualization, investigation, writing – original draft, methodology, writing – review and editing, formal analysis, data curation. **Johanna Messerly**: conceptualization, investigation, writing – original draft, writing – review and editing, methodology, formal analysis, data curation.

## Funding

The authors have nothing to report.

## Data Availability

The data that support the findings of this study are available from the corresponding author upon reasonable request.
